# A Naturally Occurring Single Nucleotide Polymorphism in the *Salmonella* SPI-2 Type III Effector *srfH*/*sseI* Controls Early Extraintestinal Dissemination

**DOI:** 10.1371/journal.pone.0045245

**Published:** 2012-09-18

**Authors:** Joshua M. Thornbrough, Micah J. Worley

**Affiliations:** 1 Department of Biology, University of Louisville, Louisville, Kentucky, United States of America; 2 Department of Microbiology and Immunology, University of Louisville, Louisville, Kentucky, United States of America; Purdue University, United States of America

## Abstract

CD18 expressing phagocytes associated with the gastro-intestinal (GI) epithelium can shuttle *Salmonella* directly into the bloodstream within a few minutes following microbial ingestion. We have previously demonstrated that *Salmonella* controls the CD18 pathway to deeper tissue, manipulating the migratory properties of infected cells as an unappreciated component of its pathogenesis. We have observed that one type III effector, SrfH (also called SseI) that Salmonella secretes into infected phagocytes manipulates the host protein TRIP6 to stimulate their migration. Paradoxically, SrfH was shown in another study to subvert a different host protein, IQGAP1, in a manner that inhibits the productive motility of such cells, perhaps to avoid interactions with T cells. Here, we resolve the discrepancy. We report that one naturally occurring allele of *srfH* promotes the migration of infected phagocytes into the bloodstream, while another naturally occurring allele that differs by only a single nucleotide polymorphism (SNP) does not. This SNP determines if the protein contains an aspartic acid or a glycine residue at position 103 and may determine if SrfH binds TRIP6. SrfH Gly103 is a rare allele, but is present in the highly invasive strain *Salmonella enterica* serovar Typhimurium UK-1 (stands for universal killer). It is also present in the genome of the only sequenced strain belonging to the emerging pandemic *Salmonella enterica* serovar 4, [5],12,i:-, which is frequently associated with septicemia. Finally, we present evidence that suggests that Gifsy-2, the bacteriophage upon which *srfH* resides, is present in a clinical isolate of the human-specific pathogen, *Salmonella enterica* serovar Typhi. These observations may have interesting implications for our understanding of *Salmonella* pathogenesis.

## Introduction

*Salmonella* is a bacterial pathogen of humans and both warm and cold-blooded animals that can actively invade host cells and proliferate within ones that are normally microbicidal. *Salmonella* is a major public health problem, which leads to more than three million deaths per year [1]. *Salmonella enterica* serovar Typhimurium (*S.* Typhimurium) usually causes a self-limiting gastroenteritis in humans, but septicemia associated with non-typhoidal *Salmonella* is a growing public health problem, which can affect otherwise healthy individuals, and is especially troublesome in certain immunodeficient people, including those infected with HIV. The closely related *Salmonella enterica* serovar Typhi (*S.* Typhi) causes typhoid fever, a systemic disease. In addition to public health concerns, *S.* Typhimurium is also studied because it is a model pathogen without parallel for dissecting basic pathogenic processes, due to its genetic tractability and the availability of excellent murine models of infection. *S.* Typhimurium produces an acute, systemic disease in BALB/c mice and produces a chronic carrier state in wild type 129X1/Sv mice, similar to the two types of disease observed with *S*. Typhi infections of humans. The acute phase is often fatal whereas in the carrier state the infection becomes largely asymptomatic, but the bacteria can be shed from internal organs, potentially for the lifetime of the host.

*S. enterica* utilizes two independent type III secretion systems encoded by *Salmonella* pathogenicity islands 1 (SPI-1) and 2 (SPI-2) to promote its virulence. The bacteria utilize SPI-1 in the gastrointestinal (GI) stage of disease to invade cells and to invoke the inflammatory response [2]–[4]. *S. enterica* is traditionally thought to only deploy SPI-2 in the systemic phase of disease, to facilitate intracellular survival and growth [5]–[7]. However, it was shown in one study that *S.* Typhimurium expresses SPI-2 associated genes in as little as 15 minutes within the GI tract, prior to penetrating the intestine [8].

SrfH was first identified as a gene regulated by the SPI-2 encoded transcription factor SsrB, even though SrfH is located outside of SPI-2 [9]. It was subsequently shown to be a SPI-2 secreted type III effector [10]. SrfH was reported to facilitate the rapid penetration of the bloodstream by infected phagocytes [11]. Another seemingly contradictory study demonstrated that SrfH repressed the productive motility of such cells [12]. The former study utilized *S.* Typhimurium 14o28s. The latter study showed that *in vitro*, SrfH from a different strain, *S.* Typhimurium SL1344, causes infected macrophages and dendritic cells to migrate aberrantly, not productively responding to chemotactic gradients composed of microbial components or CCL19 respectively [12]. CCL19 gradients normally facilitate dendritic cell-T cell interactions. This behavior requires productively interacting with IQGAP1 via a critical cysteine residue at position 178 [12]. Mutating this residue to alanine does not affect SrfH secretion or subsequent binding to IQGAP1, but blocks a productive interaction. A productive SrfH/IQGAP1 interaction increases bacterial numbers in both the spleen and liver at 45 days post-infection of wild type mice [12]. It was shown that when roughly the same number of *S.* Typhimurium SL1344 bacteria or a *srfH* mutant were present in the spleen, the presence of an intact copy of *srfH* correlated with lower numbers of CD4^+^ T cells. It was concluded that SrfH interferes with the productive motility of the phagocytes harboring it to suppress an immune response. In this study, the authors reported that SrfH does not bind TRIP6 [12].

We demonstrate here that the *srfH* alleles from the *S.* Typhimurium strains 14028s and SL1344 are not identical. A single base pair (bp) difference between the two alleles produces proteins containing different amino acids at position 103. The SL1344 SNP eliminates TRIP6 binding in a yeast two-hybrid assay as well as the early travel of infected phagocytes to the bloodstream. This study provides a remarkable example of naturally occurring allelic variants differing by only a SNP, having seemingly antagonistic effects on the same host cell process.

## Results

### The Two *srfH* Alleles are not Identical

We have sequenced multiple, independent PCR products containing the *srfH* allele from an isolate of *S.* Typhimurium SL1344 obtained from the *Salmonella* genetic stock center and determined that it is one bp different than all three independent Genbank versions of *S.* Typhimurium 14028s *srfH* (Fig. S1), which are all identical (Sam Miller’s group (AF236075.1), Lionello Bossi’s group (AF254763) and ourselves (AF231470.2)). This SNP changes Gly103 (relative to 14028s) to Asp103 (SL1344). In support of our observation, the published *S.* Typhimurium 14028s [13] and unpublished *S.* Typhimurium SL1344 (http://www.sanger.ac.uk/Projects/Salmonella/) genome sequences contain this SNP.

### SrfH Gly103 but not SrfH Asp103 Promotes Early Travel to the Bloodstream

**Figure 1:**
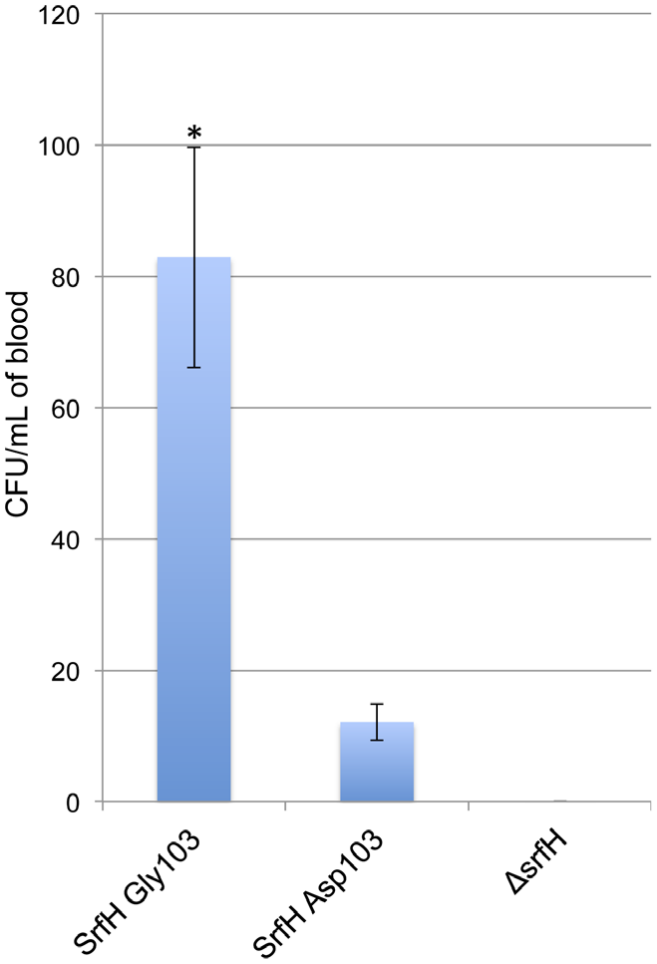
SrfH Gly103 facilitates rapid bacterial penetration of the bloodstream whereas SrfH Asp103 does not. Significantly more bacteria were recovered from the blood of mice orally inoculated thirty minutes earlier with *S.* Typhimurium SL1344 SrfH Gly103 than with *S.* Typhimurium SL1344 SrfH Asp103 (p-value <0.05). The number of CFUs recovered from the blood of mice infected with *S*. Typhimurium SL1344 SrfH Asp103 was not significantly different from zero (p–value = 0.06). No bacteria could be recovered from the blood of mice infected with *S.* Typhimurium SL1344 Δ*srfH* (p-value as compared to SrfH Gly103 = 0.01). P-values were determined with a two-tailed Student’s t test assuming unequal variance as determined by an f test. Two outliers, one from a single mouse out of 32 infected with *S.* Typhimurium SL1344 SrfH Asp103 and one from a single mouse out of 32 infected with *S.* Typhimurium SL1344 SrfH Gly103, in which tens of thousands of CFUs were recovered, presumably attributable to minor esophageal abrasions being introduced with the gavage needle were excluded. Error bars depict the standard error of the mean.

*S*. Typhimurium travels from the GI tract to the bloodstream exclusively inside of CD18^+^ phagocytes in the first 30 minutes following oral inoculation [11], [14]. To determine if SrfH Asp103, is as capable as SrfH Gly103 in promoting rapid travel to the bloodstream in such cells, we generated two new strains. In one, we deleted the entire *srfH* open reading frame from *S.* Typhimurium SL1344. In another, with allelic exchange, we generated a *S*. Typhimurium SL1344 knock-in mutant that carries the SrfH Gly103 allele instead of its native *srfH* allele. Throughout the genome, the parent strain and the derivative differ by only a SNP. To assess the impact of residue 103 of SrfH on virulence in an animal model, we introduced either wild-type *S.* Typhimurium SL1344 SrfH Asp103 or the derivative *S*. Typhimurium SL1344 SrfH Gly103, or the *srfH* deletion into BALB/c mice by oral gavage. Thirty minutes after inoculation, significantly more colony forming units (CFUs) were recovered from the blood of the mice infected with the knock-in mutant than from the blood of mice infected with the parent strain (p-value <0.05) (Fig. 1). This experiment was performed on groups of eight mice on four independent occasions. Although a few wild type *S.* Typhimurium SL1344 bacteria were recovered, the number was not significantly different from zero (p-value = 0.06). No bacteria could be recovered from the blood of any mice infected with the *srfH* deletion strain. The *srfH* deletion strain was similarly tested on groups of eight mice, but only on three independent occasions. The difference in the number of blood-borne bacteria recovered from the mice infected with the knock-in mutant versus the *srfH* deletion strain was highly significant (p-value = 0.01).

### Secretion and Stability of the SrfH Variants

**Figure 2:**
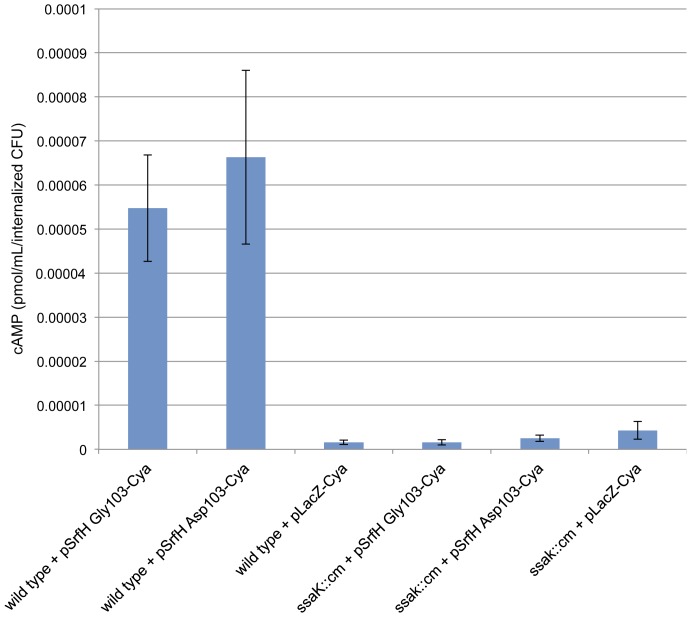
Both *srfH* alleles encode proteins that are secreted similarly. BMDMs were infected with *S*. Typhimurium SL1344 or *S*. Typhimurium SL1344 *ssak*::*cm* (a mutant defective in all SPI-2 secretion) expressing fusion proteins composed of one or the other variants or LacZ (a negative control) fused to Cya. At six hours post-infection, the infected BMDMs were lysed and cAMP levels measured by ELISA. There was no significant difference in the secretion of the two variants. This experiment was performed in triplicate on two independent occasions. Error bars represent the standard error of the mean.

Even though residue 103 is outside of the secretion signal for SrfH [10], one possible explanation for the different results obtained with the two *srfH* alleles is that the encoded proteins are secreted into phagocytes differently. To address this possibility, we generated three fusion proteins. One was composed of SrfH Asp103 fused to the adenylate cyclase gene (Cya) from *Bordetella pertussis*, one was composed of SrfH Gly103 fused to Cya, and the final one was composed of LacZ fused to Cya (to serve as a negative control). *B. pertussis* Cya only generates cAMP in the presence of calmodulin, which is not found in bacteria. This allows for very sensitive and precise detection of protein secretion by *S.* Typhimurium into infected macrophage cytosol [10], [15]. Bone marrow derived macrophages (BMDMs) were separately infected with bacteria expressing the three fusion proteins and cAMP levels measured by ELISA. As can be seen in Figure 2, the two *srfH* alleles are secreted by SPI-2 into infected macrophage cytosol similarly. Another explanation for the results of the murine dissemination experiment might be that SrfH Asp103 is unstable within mammalian cells. This seems unlikely as a different study observed SrfH Asp103-mediated phenotypes over extended periods of time within BMDMs and dendritic cells [12].

### SrfH Gly103 Binds TRIP6 in a Yeast Two-hybrid Assay but SrfH Asp103 does not

**Figure 3:**
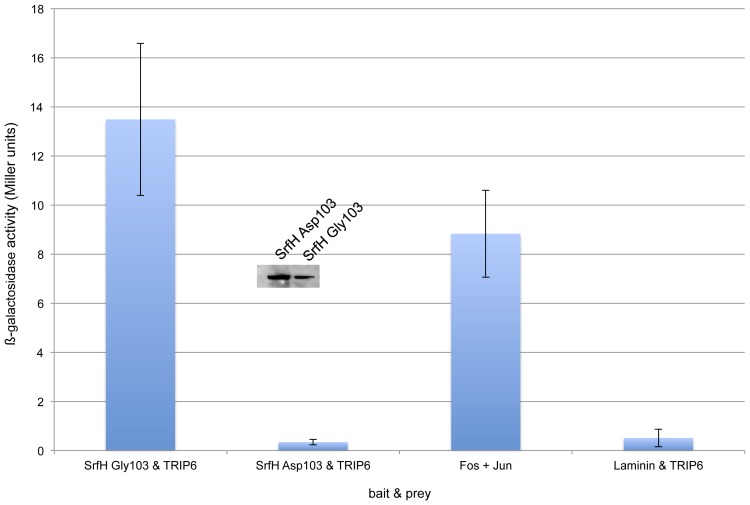
SrfH Gly103 but not SrfH Asp103 binds TRIP6 in a yeast two-hybrid assay. ß-galactosidase assays were used to identify interactions between the two SrfH variants and TRIP6 in a yeast two-hybrid assay. Laminin served as an irrelevant negative control while Fos and Jun served as a strong positive control. SrfH Gly103 readily bound TRIP6, while SrfH Asp103 did not. This experiment was performed on two independent occasions. Error bars depict the standard deviation. Equal amounts of total protein from lysates of the two yeast strains were separated by SDS-PAGE and then fusion proteins of the correct size detected in a Western blot revealing that both SrfH variants can be stably expressed within yeast.

Considering that SrfH Gly103 promotes the spread of infected phagocytes into the bloodstream and SrfH Asp103 does not, we next tested if the SL1344 SNP interfered with TRIP6 binding. To test this possibility, we cloned SrfH Gly103 and SrfH Asp103 into the bait vector of a yeast two-hybrid system, and cloned full-length TRIP6 into the prey vector. Both bait constructs were sequenced to exclude the possibility that an error was inadvertently introduced in the PCR reactions. We introduced both constructs into a yeast strain engineered to use ß-galactosidase activity as readout for bait/prey binding. As can be seen in Figure 3, SrfH Asp103 is unable to bind TRIP6 in this assay, while SrfH Gly103 is, even though both alleles can be expressed stably in yeast. This result is only suggestive as yeast two-hybrid assays can produce both false positives and false negatives. A more definitive answer as to whether or not SrfH Gly103 binds TRIP6 awaits the results of ongoing biochemical clarification.

### Natural Distribution of the Two Alleles

We analyzed *srfH* sequences from all of the completed, publicly available *Salmonella* genomes and found that SrfH Gly103 is a very rare allele, being present in only three *Salmonella* strains in addition to *S.* Typhimurium 14028s. It is present in *S*. Typhimurium strain UK-1, *S. enterica* serovar Newport strain SL254 and in *S. enterica* serovar 4, [5],12:i:- (*S.* 4, [5],12:i:-) strain CVM23701. *S*. Typhimurium UK-1 is interesting in that it is the most invasive *Salmonella* strain commonly studied [16], [17]. Attenuated mutants of this strain have accordingly been used extensively in vaccine development [18]. *S*. 4, [5],12:i:- is an emerging pandemic serovar. It was only rarely associated with disease in the mid-1990s but is now one of the most common serotypes associated with human disease in many countries around the world [19]. It was the fourth most common serovar associated with human disease in the European Union in 2006 and infections caused by it are often severe [19]. An outbreak in New York city in 1998 resulted in a 70% hospitalization rate [20]. The significance of this serovar is heightened by the fact that its members are usually multi-drug resistant [21]. Interestingly, in Thailand and Brazil it is frequently associated with septicemia [22]–[24]. In fact, in São Paulo Brazil, between 1991 and 2000, 25% of human infections by *S.* 4, [5],12:i: produced septicemia [24].

### *srfH* and Perhaps Gifsy-2 are Present in a Clinical Isolate of *S.* Typhi

*srfH* is not present in the laboratory strain *S.* Typhi Ty2 or in strain CT18. One report however presented a zoo-blot, which indicated that there was a *srfH* homolog in a strain of *S.* Typhi isolated in Dakar in 1988 [25]. We confirmed this report by PCR amplifying *srfH* from *S.* Typhi Dakar with primers designed to match the *srfH* allele of *S.* Typhimurium (Fig. S2). The *srfH* allele in *S*. Typhi Dakar encodes Asp103. We next attempted to PCR amplify two other genes believed to be specific to Gifsy-2: *gtgA* and *sodCI.* Both genes can be readily PCR amplified from *S.* Typhi Dakar (Fig. S2).

## Discussion

This study describes a unique effect of *srfH* allelic variants on *Salmonella* pathogenesis and provides us with new insights into the complex, sometimes strain specific, interactions of pathogens with host cells. Remarkably, a SNP is all that separates the two *srfH* alleles, causing them to have seemingly antagonistic effects on the migration of infected phagocytes. We have demonstrated that a glycine versus an aspartic acid residue at position 103 of SrfH allows *S*. Typhimurium to rapidly enter the bloodstream. As all such bacteria are within CD18 expressing phagocytes at early time points [11], [14], our results indicate that SrfH Gly103 can stimulate the migration of these cells, likely through the subversion of TRIP6.

It is interesting to speculate about the mechanism through which SrfH Gly103 promotes the migration of infected phagocytes into the bloodstream. TRIP6 is a member of a sub-family of adaptor proteins that localize to focal adhesions upon activation and govern adhesion and motility. This family of proteins contains amino terminal proline rich regions and three copies of the zinc finger LIM domain motif at their carboxyl terminal ends, which function as molecular platforms to mediate protein-protein interactions [26]. The coupling of the focal adhesion adaptor protein p130^cas^ (CAS) with the focal adhesion regulatory molecule Crk serves as a ‘molecular switch’ for the induction of migration through the Rac signaling pathway [27], [28]. Interestingly, TRIP6 is capable of mediating CAS/Crk coupling [29], and host directed S-palmitoylation of residue 9 of SrfH targets the protein to the plasma membrane of host cells [30]. Thus, one model for the ability of SrfH Gly103 to accelerate cellular motility is that SrfH binds TRIP6 in the cytosol and enriches it within focal adhesions where CAS and Crk are in sufficient quantities to be coupled. If this model were correct, SrfH Gly103 would only be causing a kinetic change in the migration of infected phagocytes and this implies that a chemokine receptor is up regulated either indirectly as a result of infection, or perhaps more likely, deliberately by the microbe. As attractive as this model may be, we cannot of course exclude at this point the possibility that TRIP6 binding interferes with IQGAP1 binding and/or manipulation.

Much further molecular epidemiological work will be required to answer several lingering and intriguing questions about the natural distribution of the two *srfH* alleles. It is noteworthy that SrfH Gly103 is a rare allele, but is present in two highly invasive *Salmonella* strains, as we observed that this residue is important for the extraintestinal dissemination of *Salmonella* at early time points in mice. Whether or not SrfH Gly103 contributes to the high invasiveness of *S*. Typhimurium UK-1 and *Salmonella* 4, [5],12:i:- CVM23701 needs to be determined. It will also be important in future work to determine the distribution of the two alleles in *S.* 4, [5],12:i:- and perhaps, if supportive data is uncovered, to address the intriguing possibility that the acquisition of SrfH Gly103 by members of this serovar influenced the rate of septicemia associated with it and its pandemic rise over the last 15 years. It is possible that SrfH Gly103 is an emerging allele that will become more prevalent over time, but it could also of course be an anti-virulence factor. Unfortunately, the *srfH* alleles appear to be clonal outside of residue 103 making it difficult to determine which of the two alleles is newer.

What effects, if any, that *srfH*, *gtgA* and *sodCI* have on *S*. Typhi pathogenesis warrant consideration. Inactivating *gtgA* in *S*. Typhimurium does not have a detectable impact on virulence in the mouse model of typhoid fever [31]; however, it was identified along with *srfH* as contributing to virulence in the murine, long-term *Salmonella* infection model [32]. Its function remains to be determined. SodCI on the other hand has a well-established role in promoting *S.* Typhimurium pathogenesis. Superoxide dismutases are necessary to protect bacteria from reactive oxygen species generated from the phagocytic respiratory burst. *S.* Typhimurium 14028s produces two periplasmic Cu/Zn superoxide dismutases. SodCII is encoded on the chromosome, outside of Gifsy-2, and has an ortholog in *Escherichia coli*. Its absence does not confer a virulence defect. However, *sodCI* provides a 7 to 10 fold survival advantage [33], [34]. SodCI in fact, possesses one of highest catalytic rates ever measured for superoxide dismutases [35]. In future work, it will be interesting to assess if SrfH assists *S*. Typhi Dakar in manipulating the migratory properties of infected phagocytes. It will be of further interest to determine, what, if any, effect SodCI has on the ability of *S.* Typhi Dakar to survive within macrophages.

It is also interesting to consider the origins of *gtgA*, *sodCI* and *srfH* in *S.* Typhi Dakar. These three genes could have become associated with mobile elements other than Gifsy-2 and acquired, potentially, independently of each other by *S.* Typhi Dakar; however, the simplest explanation would of course be that *S.* Typhi Dakar somehow acquired Gifsy-2. Gifsy-2 is a fully functional bacteriophage, capable of excising itself from the *S.* Typhimurium chromosome and transferring itself to non-immune recipient *Salmonella* strains [31]. However, the attachment site in most strains of *S*. Typhi is occupied by a related phage that confers immunity to Gifsy-2 [31]. Thus, more work will be required to determine if Gifsy-2 is indeed present in *S.* Typhi Dakar, and if so, if it has managed to replace the resident prophage, or alternatively has integrated into the chromosome somewhere other than at its normal attachment site.

In addition to shedding light on one pathway through which non-typhoidal *Salmonella* can enter the bloodstream, studying SrfH may also have implications for understanding *S*. Typhi pathogenesis. While *srfH* is not present in the genomes of all strains of *S*. Typhi, most of them probably have *srfH* functional analog(s), and there will likely be additional strains found to encode SrfH, perhaps including ones that possess Gly103. Pathogen directed host cell migration might prove to be a common feature of invasive pathogens of humans. Thus, unraveling on a molecular level exactly how SrfH subverts host cell motility to promote virulence will perhaps not only enhance our knowledge of *Salmonella* pathogenesis but also reveal some of the underlying shared logic through which pathogens manipulate host cells.

## Materials and Methods

### Ethics Statement

Animals were housed, cared for, and used strictly in accordance with the USDA regulations and the NIH guide for the care and use of laboratory animals (NIH publication no. 85–23, 1985). The University of Louisville is fully accredited by the American Association for the Accreditation of Laboratory Animal Care. A full-time, specialty-trained veterinarian directs the program of animal care. The protocol was approved by the University of Louisville Institutional Animal Care and Use Committee (protocol # 11032). All reasonable efforts were made to alleviate discomfort.

### General Methods

All molecular biology and genetic manipulations were performed with established protocols [36], [37]. It was determined that SrfH Asp103 does not bind TRIP6 with the Hybrid Hunter yeast two-hybrid assay kit (Invitrogen, Carlsbad, CA), following the manufacturer’s directions, which can be found online at: http://www.tcd.ie/Genetics/staff/Noel.Murphy/recombinant %20dna%20ge4021/hybridman.pdf. ß-Gal assays were performed on cultures grown with rigorous shaking at 30°C using a protocol available at http://labs.fhcrc.org/gottschling/Yeast%20Protocols/Bgal.html, which was adapted from Current Protocols in Molecular Biology [36]. It was determined that both *srfH* alleles could be stably expressed in yeast by lysing the yeast according to the kit’s directions and then subjecting the lysates to SDS-PAGE and visualizing a hybrid protein of the correct molecular weight after performing a Western blot with an anti-LexA antibody (Invitrogen). LexA was the DNA binding domain that SrfH was fused to in the yeast two-hybrid assay.

### Mice, Cell Culture and Bacterial Strains

Four-week-old female Balb/cJ mice were obtained from Jackson labs. (Bar Harbor, ME). Bone marrow was harvested and differentiated into primary macrophages, and cultivated in RPMI (ATCC, Manassas, VA), supplemented with 20% FBS (Sigma, St. Louis, Mo) and 30% L929 (ATCC) conditioned media as previously described [38]. *S.* Typhimurium SL1344 was obtained from the *Salmonella* genetic stock center (*Salmonella* genetic stock center # 438), originally described in Hoiseth and Stocker [39]. This was the parent strain used in this study which is also the same one used by McLaughlin et. al [12]. The entire *srfH* open reading frame was deleted with the method of Datsenko and Wanner [40]. To generate a SrfH Gly103 knock-in mutant in an SL1344 background, the SrfH Gly103 allele was PCR amplified from the chromosome of *S.* Typhimurium 14028s, treated with T4 polynucleotide kinase, and blunt end ligated into the suicide plasmid pRE112 [41], which had been digested with SmaI and dephosphorylated, yielding pMJW2150. This construct was recovered in the *Esherichia coli* strain MFDpir, which has been engineered to allow the propagation of suicide plasmids [42]. pRE112 contains the counter-selectable marker *sacB1* as well as a chloramphenicol cassette. *S.* Typhimurium SL1344 was conjugated with MFDpir + pMJW2150 and merodiploids at the *srfH* locus recovered on agar plates supplemented with chloramphenicol, but lacking diaminopimelic acid (DAP). The chloramphenicol killed the *S*. Typhimurium SL1344 cells which did not become merodiploids and the lack of DAP prevented MFDpir cells from growing. Ten merodiploids were passaged serially three times in LB broth without DAP or antibiotics to give them a chance to resolve the duplicated allele. They were then grown to mid-exponential phase and plated on LB-agar plates without NaCl, containing 5% sucrose. The sucrose killed the remaining merodiploids. The strains in which SrfH Gly103 had replaced SrfH Asp103 were identified by PCR and sequencing. The new allele was then PCR amplified from one such strain, MJW2163, with oligonucleotides that flanked the exchanged material, and the entire area sequenced to ensure that no secondary mutations had been inadvertently introduced.

### Mouse Dissemination Assay

Mice were orally administered an inoculum of 1×10^9^ bacteria suspended in 100 µl of phosphate buffered saline. Food was withheld for 12 hours prior to infection. Thirty minutes after oral infection, mice were euthanized with CO_2_ and blood recovered by heart puncture with a 25 G needle attached to a 1 mL syringe. 400 µl of blood from each mouse was collected in microtubes on ice containing 25 units of heparin sodium salt (Sigma) in 50 µl of water to prevent coagulation, as well as 50 µl of 10% Triton X-100 to lyse host cells. The tubes were incubated at 4**°**C with end over end rotation on a rotisserie for ten minutes. 200 µl of blood was then plated on xylose lysine deoxycholate agar (XLD) plates supplemented with 200 µg/mL streptomycin. XLD plates are selective for enteric bacteria and *S*. Typhimurium SL1344 is naturally resistant to streptomycin. The following day CFUs were enumerated and the number of bacteria per milliliter of blood calculated.

### Adenylate Cylase Fusion Secretion Assay

The entire *srfH* open reading frames from *S.* Typhimurium strains 14028s and SL1344 were PCR amplified and cloned into a SmaI digested pMJW1753, which contains bp 4 to 1233 of *B. pertussis cya* [43]. The resulting fusion proteins were expressed in *S*. Typhimurium SL1344 as well as a derivative in which the *ssaK* allele was replaced with a chloramphenicol cassette with the method of Datsenko and Wanner [40], rendering it incapable of SPI-2 mediated secretion. BALB/c BMDMs were seeded into 96 well plates (4×10^4^ cells/well) and twenty-four hours later infected at a multiplicity of infection of 50 for one hour as part of a standard gentamicin protection assay. The infected macrophages were lysed with 0.1 M HCL at 6 hours post-infection and cAMP levels measured by ELISA (Enzo Life Sciences, Ann Arbor, MI), following the manufacturer’s directions, using the acetylation option. A duplicate plate was lysed with 1% Triton X-100 and CFU recovered on agar plates.

### Detection of Gifsy-2 Specific Genes in *S.* Typhi Dakar

*S.* Typhi Dakar was obtained from the *Salmonella* genetic stock center (*Salmonella* genetic stock center # 3036). The oligonucleotides used were: *srfH* forward ATGCCCTTTCATATTGGAAGCGG, *srfH* reverse CTGATGGGCTGAAACATCAAACAC, *gtgA* forward CCAGAACACCAAGATAATCCTTCG, *gtgA* reverse TCCAGGTCCTCATCAGTTACGG, *sodCI* forward TGTTCAGCAATGGCAGAGAATACC, *sodCI* reverse CAATGACACCACAGGCAAAACG. The three genes were PCR amplified with PfuUltra II DNA polymerase (Agilent technologies, Santa Clara, CA) using the following cycling conditions: 95**°**C for 10 minutes, then 35 cycles of 95**°**C for 30 seconds, 55**°**C for 30 seconds and then 72**°**C for 30 seconds followed by a 10 minute incubation at 72**°**C.

## Supporting Information

Figure S1 **Sequencing chromatograms of internal regions of the** ***srfH*** **open reading frames.** A) strain 14028s and B) strain SL1344. The arrows indicate the G A SNP.
(TIF)

Figure S2 **Three Gifsy-2-specific genes are present in a clinical isolate of** ***S. typhi.*** Multiplex PCR with primers specific to internal regions of the *S.* Typhimurium strain 14028s *srfH*, *sodCI*, and *gtgA* alleles were PCR amplified from the genome of an isolate of *S.* Typhi Dakar. The first lane contains a DNA ladder with bands corresponding to (from top to bottom) 1 Kb, 850 bp, 650 bp, 500 bp, 400 bp, 300 bp, 200 bp and finally 100 bp. The next lane contained genomic DNA from *S.* Typhi Dakar. The final lane was a no template, negative control. The predicted sizes of the PCR products are 708 bp for *srfH*, 481 bp for *sodCI*, and 329 bp for *gtgA*.
(TIF)

## References

[1] Pui CF, Wong WC, Chai LC, Tunung R, Jeyaletchumi P, et al (2011) Salmonella: A foodborne pathogen. Int Food Res J 18: 465–473.

[2] Groisman EA, Ochman H (1993) Cognate gene clusters govern invasion of host epithelial cells by Salmonella typhimurium and Shigella flexneri. Embo J 12: 3779–3787. 8404849 10.1002/j.1460-2075.1993.tb06056.x PMC413660

[3] Galyov EE, Wood MW, Rosqvist R, Mullan PB, Watson PR, et al (1997) A secreted effector protein of Salmonella dublin is translocated into eukaryotic cells and mediates inflammation and fluid secretion in infected ileal mucosa. Mol Microbiol 25: 903–912. 9364916 10.1111/j.1365-2958.1997.mmi525.x

[4] Hobbie S, Chen LM, Davis RJ, Galan JE (1997) Involvement of mitogen-activated protein kinase pathways in the nuclear responses and cytokine production induced by Salmonella typhimurium in cultured intestinal epithelial cells. J Immunol 159: 5550–5559. 9548496

[5] Cirillo DM, Valdivia RH, Monack DM, Falkow S (1998) Macrophage-dependent induction of the Salmonella pathogenicity island 2 type III secretion system and its role in intracellular survival. Mol Microbiol 30: 175–188. 9786194 10.1046/j.1365-2958.1998.01048.x

[6] Hensel M, Shea JE, Waterman SR, Mundy R, Nikolaus T, et al (1998) Genes encoding putative effector proteins of the type III secretion system of Salmonella pathogenicity island 2 are required for bacterial virulence and proliferation in macrophages. Mol Microbiol 30: 163–174. 9786193 10.1046/j.1365-2958.1998.01047.x

[7] Ochman H, Soncini FC, Solomon F, Groisman EA (1996) Identification of a pathogenicity island required for Salmonella survival in host cells. Proceedings of the National Academy of Sciences of the United States of America 93: 7800–7804. 8755556 10.1073/pnas.93.15.7800 PMC38828

[8] Brown NF, Vallance BA, Coombes BK, Valdez Y, Coburn BA, et al (2005) Salmonella pathogenicity island 2 is expressed prior to penetrating the intestine. PLoS Pathog 1: e32. 16304611 10.1371/journal.ppat.0010032 PMC1287911

[9] Worley MJ, Ching KH, Heffron F (2000) Salmonella SsrB activates a global regulon of horizontally acquired genes. Mol Microbiol 36: 749–761. 10844662 10.1046/j.1365-2958.2000.01902.x

[10] Miao EA, Miller SI (2000) A conserved amino acid sequence directing intracellular type III secretion by Salmonella typhimurium. Proc Natl Acad Sci U S A 97: 7539–7544. 10861017 10.1073/pnas.97.13.7539 PMC16581

[11] Worley MJ, Nieman GS, Geddes K, Heffron F (2006) Salmonella typhimurium disseminates within its host by manipulating the motility of infected cells. Proc Natl Acad Sci U S A 103: 17915–17920. 17095609 10.1073/pnas.0604054103 PMC1635543

[12] McLaughlin LM, Govoni GR, Gerke C, Gopinath S, Peng K, et al (2009) The Salmonella SPI2 effector SseI mediates long-term systemic infection by modulating host cell migration. PLoS Pathog 5: e1000671. 19956712 10.1371/journal.ppat.1000671 PMC2777311

[13] Jarvik T, Smillie C, Groisman EA, Ochman H (2010) Short-term signatures of evolutionary change in the Salmonella enterica serovar typhimurium 14028 genome. J Bacteriol 192: 560–567. 19897643 10.1128/JB.01233-09 PMC2805332

[14] Vazquez-Torres A, Jones-Carson J, Baumler AJ, Falkow S, Valdivia R, et al (1999) Extraintestinal dissemination of Salmonella by CD18-expressing phagocytes. Nature 401: 804–808. 10548107 10.1038/44593

[15] Miao EA, Scherer CA, Tsolis RM, Kingsley RA, Adams LG, et al (1999) Salmonella typhimurium leucine-rich repeat proteins are targeted to the SPI1 and SPI2 type III secretion systems. Mol Microbiol 34: 850–864. 10564523 10.1046/j.1365-2958.1999.01651.x

[16] Luo Y, Kong Q, Yang J, Mitra A, Golden G, et al (2012) Comparative Genome Analysis of the High Pathogenicity Salmonella Typhimurium Strain UK-1. PLoS ONE 7: e40645. 22792393 10.1371/journal.pone.0040645 PMC3391293

[17] Luo Y, Kong Q, Yang J, Golden G, Wanda SY, et al (2011) Complete genome sequence of the universal killer Salmonella enterica Serovar Typhimurium UK-1 (ATCC 68169). J Bacteriol 193: 4035–4036. 21622747 10.1128/JB.05224-11 PMC3147501

[18] Zhang X, Kelly SM, Bollen W, Curtiss R, 3rd (1999) Protection and immune responses induced by attenuated Salmonella typhimurium UK-1 strains. Microb Pathog 26: 121–130. 10089152 10.1006/mpat.1998.0245

[19] Switt AI, Soyer Y, Warnick LD, Wiedmann M (2009) Emergence, distribution, and molecular and phenotypic characteristics of Salmonella enterica serotype 4,5,12:i. Foodborne Pathog Dis 6: 407–415. 19292687 10.1089/fpd.2008.0213 PMC3186709

[20] Agasan A, Kornblum J, Williams G, Pratt CC, Fleckenstein P, et al (2002) Profile of Salmonella enterica subsp. enterica (subspecies I) serotype 4,5,12:i:- strains causing food-borne infections in New York City. J Clin Microbiol 40: 1924–1929. 12037044 10.1128/JCM.40.6.1924-1929.2002 PMC130705

[21] Hopkins KL, Kirchner M, Guerra B, Granier SA, Lucarelli C, et al (2010) Multiresistant Salmonella enterica serovar 4,[5],12:i:- in Europe: a new pandemic strain? Euro Surveill 15: 19580. 20546690

[22] Pornruangwong S, Sriyapai T, Pulsrikarn C, Sawanpanyalert P, Boonmar S, et al (2008) The epidemiological relationship between Salmonella enterica serovar typhimurium and Salmonella enterica serovar 4,[5],12:i:- isolates from humans and swine in Thailand. Southeast Asian J Trop Med Public Health 39: 288–296. 18564715

[23] Komolpis P, Srifuengfung S, Dhiraputra C, Pingwang B (1999) Salmonella Bacteremia: Serotype Distribution and Antimicrobial Susceptibility During 1991–1995. J Infect Dis Antimicrob Agents 16: 49–52.

[24] Tavechio AT, Ghilardi AC, Fernandes SA (2004) “Multiplex PCR” identification of the atypical and monophasic Salmonella enterica subsp. enterica serotype 1,4,[5],12:i:- in Sao Paulo State, Brazil: frequency and antibiotic resistance patterns. Rev Inst Med Trop Sao Paulo 46: 115–117. 15141285 10.1590/s0036-46652004000200012

[25] Hansen-Wester I, Stecher B, Hensel M (2002) Analyses of the evolutionary distribution of Salmonella translocated effectors. Infect Immun 70: 1619–1622. 11854253 10.1128/IAI.70.3.1619-1622.2002 PMC127817

[26] Schmeichel KL, Beckerle MC (1994) The LIM domain is a modular protein-binding interface. Cell 79: 211–219. 7954790 10.1016/0092-8674(94)90191-0

[27] Kain KH, Klemke RL (2001) Inhibition of cell migration by Abl family tyrosine kinases through uncoupling of Crk-CAS complexes. J Biol Chem 276: 16185–16192. 11279004 10.1074/jbc.M100095200

[28] Klemke RL, Leng J, Molander R, Brooks PC, Vuori K, et al (1998) CAS/Crk coupling serves as a “molecular switch” for induction of cell migration. J Cell Biol 140: 961–972. 9472046 10.1083/jcb.140.4.961 PMC2141747

[29] Lai YJ, Chen CS, Lin WC, Lin FT (2005) c-Src-mediated phosphorylation of TRIP6 regulates its function in lysophosphatidic acid-induced cell migration. Mol Cell Biol 25: 5859–5868. 15988003 10.1128/MCB.25.14.5859-5868.2005 PMC1168818

[30] Hicks SW, Charron G, Hang HC, Galan JE (2011) Subcellular targeting of salmonella virulence proteins by host-mediated s-palmitoylation. Cell host & microbe 10: 9–20. 21767808 10.1016/j.chom.2011.06.003 PMC4326042

[31] Figueroa-Bossi N, Uzzau S, Maloriol D, Bossi L (2001) Variable assortment of prophages provides a transferable repertoire of pathogenic determinants in Salmonella. Mol Microbiol 39: 260–271. 11136448 10.1046/j.1365-2958.2001.02234.x

[32] Lawley TD, Chan K, Thompson LJ, Kim CC, Govoni GR, et al (2006) Genome-wide screen for Salmonella genes required for long-term systemic infection of the mouse. PLoS Pathog 2: e11. 16518469 10.1371/journal.ppat.0020011 PMC1383486

[33] Ho TD, Slauch JM (2001) Characterization of grvA, an antivirulence gene on the gifsy-2 phage in Salmonella enterica serovar typhimurium. J Bacteriol 183: 611–620. 11133955 10.1128/JB.183.2.611-620.2001 PMC94917

[34] Krishnakumar R, Craig M, Imlay JA, Slauch JM (2004) Differences in enzymatic properties allow SodCI but not SodCII to contribute to virulence in Salmonella enterica serovar Typhimurium strain 14028. J Bacteriol 186: 5230–5238. 15292124 10.1128/JB.186.16.5230-5238.2004 PMC490929

[35] Pesce A, Battistoni A, Stroppolo ME, Polizio F, Nardini M, et al (2000) Functional and crystallographic characterization of Salmonella typhimurium Cu,Zn superoxide dismutase coded by the sodCI virulence gene. J Mol Biol 302: 465–478. 10970746 10.1006/jmbi.2000.4074

[36] Ausubel FM, Brent R, Kingston RE, Moore DD, Seidman JG, et al. Current Protocols in Molecular Biology; Janssen K, editor: John Wiley & Sons, Inc.

[37] Maloy SR, Stewart VJ, Taylor RK (1996) Genetic Analysis of Pathogenic Bacteria. Cold Spring Harbor: Cold Spring Harbor Laboratory Press.

[38] Marim FM, Silveira TN, Lima DS Jr, Zamboni DS (2010) A method for generation of bone marrow-derived macrophages from cryopreserved mouse bone marrow cells. PLoS ONE 5: e15263. 21179419 10.1371/journal.pone.0015263 PMC3003694

[39] Hoiseth SK, Stocker BA (1981) Aromatic-dependent Salmonella typhimurium are non-virulent and effective as live vaccines. Nature 291: 238–239. 7015147 10.1038/291238a0

[40] Datsenko KA, Wanner BL (2000) One-step inactivation of chromosomal genes in Escherichia coli K-12 using PCR products. Proc Natl Acad Sci U S A 97: 6640–6645. 10829079 10.1073/pnas.120163297 PMC18686

[41] Edwards RA, Keller LH, Schifferli DM (1998) Improved allelic exchange vectors and their use to analyze 987P fimbria gene expression. Gene 207: 149–157. 9511756 10.1016/s0378-1119(97)00619-7

[42] Ferrieres L, Hemery G, Nham T, Guerout AM, Mazel D, et al (2010) Silent mischief: bacteriophage Mu insertions contaminate products of Escherichia coli random mutagenesis performed using suicidal transposon delivery plasmids mobilized by broad-host-range RP4 conjugative machinery. J Bacteriol 192: 6418–6427. 20935093 10.1128/JB.00621-10 PMC3008518

[43] Geddes K, Worley M, Niemann G, Heffron F (2005) Identification of new secreted effectors in Salmonella enterica serovar Typhimurium. Infect Immun 73: 6260–6271. 16177297 10.1128/IAI.73.10.6260-6271.2005 PMC1230965

