# Supplementary figures and images for: A Naturally Occurring Single Nucleotide Polymorphism in the Salmonella SPI-2 Type III Effector srfH/sseI Controls Early Extraintestinal Dissemination
Source: PLoS One. 2012 Sep 18;7(9):e45245. doi: 10.1371/journal.pone.0045245 (PMC3445477; doi:10.1371/journal.pone.0045245)

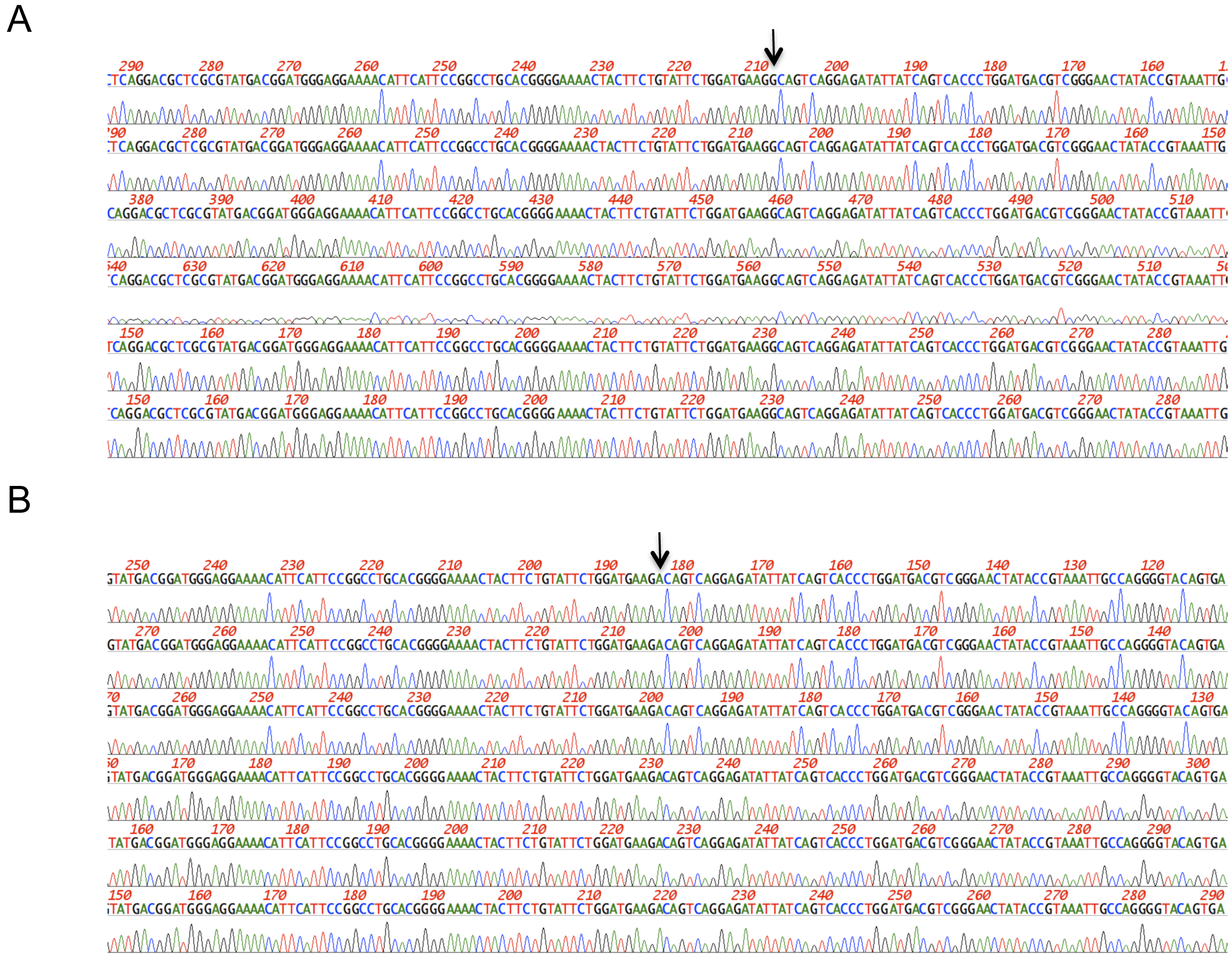

Supplement: Figure S1 — Sequencing chromatograms of internal regions of the srfH open reading frames. A) strain 14028s and B) strain SL1344. The arrows indicate the G A SNP. (TIF) [file pone.0045245.s001.tif]

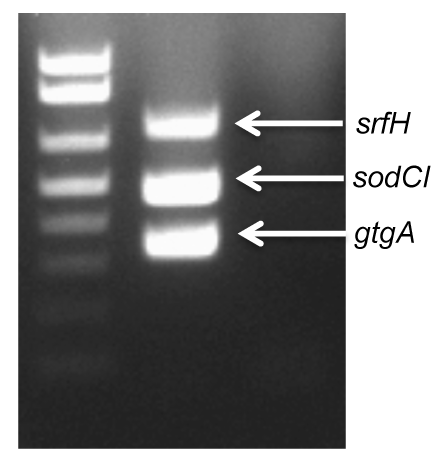

Supplement: Figure S2 — Three Gifsy-2-specific genes are present in a clinical isolate of S. typhi. Multiplex PCR with primers specific to internal regions of the S. Typhimurium strain 14028s srfH, sodCI, and gtgA alleles were PCR amplified from the genome of an isolate of S. Typhi Dakar. The first lane contains a DNA ladder with bands corresponding to (from top to bottom) 1 Kb, 850 bp, 650 bp, 500 bp, 400 bp, 300 bp, 200 bp and finally 100 bp. The next lane contained genomic DNA from S. Typhi Dakar. The final lane was a no template, negative control. The predicted sizes of the PCR products are 708 bp for srfH, 481 bp for sodCI, and 329 bp for gtgA. (TIF) [file pone.0045245.s002.tif]
